# Exposome involvement in the development of acne vulgaris

**DOI:** 10.3389/fimmu.2026.1779036

**Published:** 2026-03-17

**Authors:** Katerina Grafanaki, Daniella Bakoli Sgourou, Alexandros Maniatis, Christos C. Zouboulis

**Affiliations:** 1 Department of Dermatology-Venereology, School of Medicine, University of Patras, Patras, Greece; 2 Department of Biochemistry, School of Medicine, University of Patras, Patras, Greece; 3 Department of Dermatology, Venereology and Immunology, Universitaetsklinikum Ruppin-Brandenburg, Brandenburg Medical School Theodor Fontane and Faculty of Health Sciences Brandenburg, Neuruppin, Germany

**Keywords:** acne vulgaris, environmental and lifestyle factors, exposome, health equity and inclusion, non-coding RNAs, sebaceous gland, skin of color, social determinants of health

## Abstract

Acne vulgaris is one of the most prevalent chronic inflammatory skin diseases worldwide, characterized by marked clinical heterogeneity, fluctuating disease course, and strong sensitivity to environmental and lifestyle factors. The exposome, encompassing lifelong environmental, lifestyle, psychosocial, microbial, and intrinsic exposures, offers an integrative framework for re-conceptualizing acne as an environmentally modulated inflammatory disease. This review synthesizes external and internal exposomal drivers of acne, including pollution, radiation, climate and occupational factors, diet, smoking, cosmetics, psychosocial stress, and microbial ecosystems. We highlight the sebaceous gland as a central exposome sensor that integrates metabolic, immune, microbial, and neuroendocrine signals. Genetic susceptibility, epigenetic reprogramming, and non-coding RNA networks are key modifiers translating environmental exposures into persistent inflammatory and metabolic responses within the pilosebaceous unit. Importantly, adopting a health equity and social determinants of health (SDOH) perspective, we emphasize how structural and socioeconomic inequalities shape exposome burden, disease severity, and access to care. We propose that exposome-informed, low-cost, community-level prevention strategies, combined with evidence-based therapies, offer a pragmatic and equitable approach to acne management. Integrating molecular mechanisms with real-world and equity considerations, this framework advances understanding of acne pathophysiology and supports translation into more inclusive clinical practice.

## 1 Introduction

Acne vulgaris is among the most prevalent chronic inflammatory skin diseases worldwide, affecting both adolescents and adults and imposing a substantial psychosocial and quality-of-life burden (1–4). Its classical pathogenic model characterized by sebaceous hyperactivity, follicular hyperkeratinization, *Cutibacterium acnes (C. acnes)* colonization, and inflammation, remains foundational (5–7). However, it fails to fully explain the marked clinical heterogeneity, fluctuating disease course, and strong environmental sensitivity observed in patients with acne (8).

The sebaceous gland is now recognized as a highly dynamic neuro-immuno-endocrine organ, often referred to as the “brain of the skin,” capable of integrating endocrine, immune, microbial, and environmental signals (9, 10). Sebocytes express pattern-recognition receptors, respond to metabolic and microbial cues, and actively participate in innate immunity via cytokines, lipid mediators, and antimicrobial peptides, positioning the pilosebaceous unit as a central sensor and effector of exposomal stress (11, 12). Within the pilosebaceous unit, C. acnes functions as a commensal organism whose pathogenicity is not universal but instead depends on strain diversity, host immune context, and environmental stressors. Acne-associated C. acnes phylotypes preferentially activate Toll-like receptor-2 (TLR2), NLRP3 inflammasome signaling, and interleukin-1β (IL-1β) production in keratinocytes and sebocytes, thereby amplifying local inflammation and lesion progression (13–15).

The exposome, defined as the totality of environmental exposures from conception onward, provides an integrative framework for understanding acne as an environmentally modulated disease (16, 17). The exposome encompasses all external and internal exposures encountered across the lifespan and their cumulative impact on health and disease (18). External environmental exposures (extrinsic exposome) interact with host biology to shape the internal exposome, driving molecular, cellular, and tissue-level alterations that contribute to the onset, progression, and exacerbation of chronic inflammatory disorders such as acne (19). Environmental pollution represents a key extrinsic exposomal factor. Particulate matter (PM) and chemical pollutants induce oxidative stress, inflammatory signaling, and dysregulated lipogenesis in sebocytes, often synergizing with *C. acnes* and contributing to acne exacerbation in urban areas (20–22). Repetitive exposomal insults may sustain subclinical inflammation and sebaceous gland priming, supporting the concept of acne as a continuously evolving lesion cycle rather than a series of isolated events (23, 24).

Physical and lifestyle exposome factors further modulate the biology of acne. Ultraviolet radiation (UV) alters sebaceous lipid composition, induces oxidative stress, and modulates inflammatory pathways, challenging the historical view of UV exposure as uniformly beneficial (25). High-glycemic and lipogenic diets elevate insulin and insulin-like growth factor-1 (IGF-1) activating PI3K/Akt/mTOR while suppressing FOXO1, which promotes sebaceous lipogenesis and inflammation, with alcohol intake and specific fatty acids further amplifying these metabolic pathways (26–31). Conversely, bioactive dietary compounds include resveratrol (e.g. grapes, berries), lactoferrin (an iron-binding milk-derived protein), and tea-derived saponins (e.g. green tea, oolong tea) exert sebosuppressive and anti-inflammatory effects (32–37).

Psychosocial stress represents a critical yet often underestimated exposome domain, with catecholamines and neuroendocrine mediators influencing *C. acnes* behavior and amplifying inflammatory responses, while acne itself is strongly correlated with anxiety, depression, and reduced quality of life, reinforcing a bidirectional stress–disease cycle (4, 18, 19, 38–40). Finally, the microbial exposome, mediated by distinct *C. acnes* phylotypes and their capacity to activate inflammasome pathways, interacts dynamically with environmental and lifestyle factors, further contributing to acne development and persistence (12, 41).

**Figure 1:**
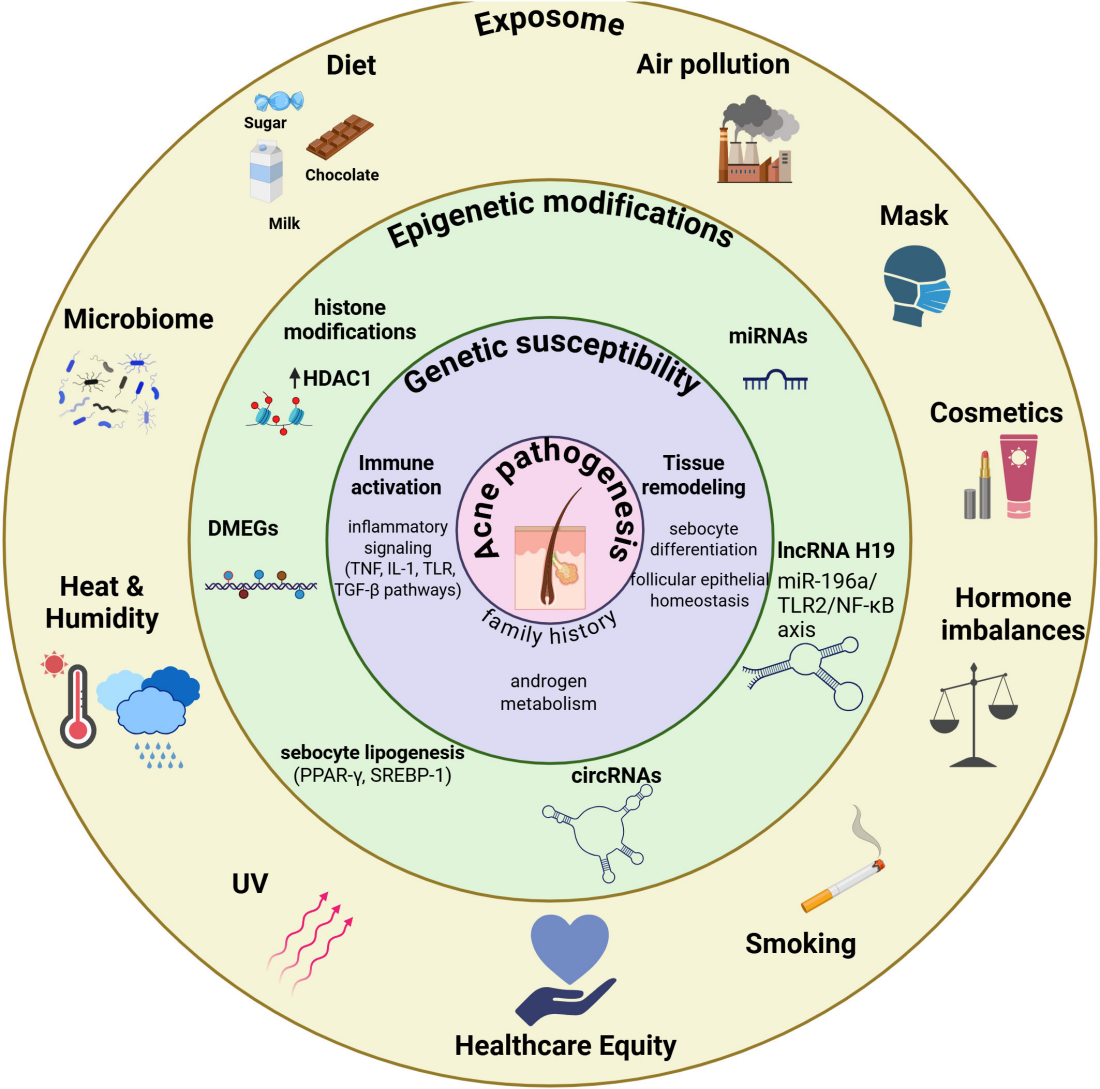
Multilayered exposome model of acne pathophysiology. Acne pathogenesis is the result of concentric and interacting determinants (figure core). The inner circle denotes genetic susceptibility, defining baseline risk. The middle circle represents epigenetic modifications that dynamically modulate gene expression in response to internal and external cues. The outer circle encompasses the extrinsic exposome, including environmental, lifestyle, microbial, psychosocial, and socioeconomic exposures. Together, these layers modulate the sebaceous gland biology and immune–metabolic crosstalk to shape acne development, severity, and persistence. Framed through a health equity lens, this model underscores the relevance of affordable, preventive, and community-level interventions to translate exposome science into equitable population health benefits. All figures were created in BioRender (31/12/2025) https://BioRender.com/ffbf58o.

In this review, we integrate these dimensions into an exposome-based model in which environmental, lifestyle, microbial, and intrinsic factors affect sebaceous gland biology and immune–metabolic crosstalk to determine acne development, severity, and persistence. From healthcare equity and inclusive lens, we integrate social determinants of health (SDOH) to support affordable, accessible and preventive acne care through community-level interventions that translate scientific advances into equitable health benefits for acne patients (**Figure 1**).

## 2 External exposome of acne vulgaris

### 2.1 Air pollution

Air pollution has emerged as a significant environmental contributor to acne vulgaris, acting through complex oxidative and inflammatory mechanisms that disturb skin homeostasis.

Population-based studies have linked exposure to air pollutants with an increased risk and severity of acne. In a Greek study, individuals working near power plants -exposed to higher levels of CO_2_, CO, SO_2_, NO_2_, and PM- had significantly greater odds of developing acne compared to those without such exposure (adjusted odds ratio [aOR] = 3.07). Moreover, severe NO_2_ exposure was associated with an even higher risk (aOR = 8.24) (42). Similarly, a large retrospective study demonstrated positive associations between PM_2.5_, PM_10_, and NO_2_ concentrations and outpatient visits for acne vulgaris, while SO_2_ showed inverse correlations (43).

PM is classified according to aerodynamic diameter, including PM_10_ (≤10 μm), PM_2.5_ (≤2.5 μm) and ultrafine particles (≤0.1 μm), with the smaller particles exhibiting greater skin penetration capacity (44, 45). Air pollutants such as PM and nitrogen oxides promote acne, primarily through oxidative stress-mediated pathways. In synergy with ultraviolet radiation, pollutants generate reactive oxygen species (ROS), which induce lipid peroxidation of sebum components like squalene, leading to comedogenic byproducts that promote follicular hyperkeratinization, barrier disruption, and inflammatory cytokine release, which favor comedogenesis and inflammatory lesion formation (46, 47).

Sebocytes are particularly susceptible to pollutant-induced stress. Experimental studies show that PM directly activates sebocytes and synergises with *C. acnes* to amplify inflammatory responses, lowering the inflammatory activation threshold within the pilosebaceous unit (48–50). A central molecular mediator linking pollution exposure to sebaceous and immune dysregulation is the aryl hydrocarbon receptor (AhR) (22). PM synergizes with *C. acnes* by amplifying TLR- and AhR-dependent signaling, increasing ROS generation, lowering inflammatory activation thresholds, and enhancing cytokine release (IL-1β, IL-6, IL-8), thus exacerbating the development of inflammatory acne lesions (49, 51). *C. acnes*–induced AhR activation modulates inflammatory and barrier-related gene expression, suggesting that pollutant-derived AhR ligands exacerbate acne by amplifying microbe-driven immune signaling. Pollution-related psychosocial and physiological stress may further modulate acne severity. Stress-induced catecholamines influence *C. acnes* behavior by enhancing bacterial growth, biofilm formation, and virulence, while concurrently amplifying host inflammatory responses through neuroimmune interactions within the pilosebaceous unit (40, 52, 53).

Clinical studies in highly polluted urban environments, including Shanghai and Mexico City, revealed pollution-associated alterations in sebum composition, reduced vitamin E and squalene levels, and increased lipid peroxidation—biochemical changes strongly implicated in acne pathogenesis (47, 54). An 8-week study in Chinese acne patients further demonstrated correlations between pollutant exposure, increased sebum secretion, and higher inflammatory and non-inflammatory lesion counts (54). Pollution-induced oxidative stress likely acts on a pre-existing vulnerable lipid milieu in acne-prone skin, characterized by oxidized squalene and reduced linoleic acid, further amplifying follicular hyperkeratinization and inflammation (55, 56).

### 2.2 Climatic factors and ultraviolet radiation in acne pathophysiology

Climatic factors, including heat, humidity, and ultraviolet radiation (UVR), significantly influence acne pathophysiology. These environmental stressors contribute to seasonal flares and acne variants such as acne tropicana, acne majorca, or tropical acne (23, 57–60).

#### 2.2.1 Heat, humidity, and seasonal variation

Elevated temperature and humidity enhance sweat and sebum secretion, promote follicular occlusion, and favor the proliferation of *C. acnes*. These factors are particularly relevant in tropical climates, where acne exacerbations are common. Seasonal variation has been consistently documented: in one study of 452 patients, over 50% reported worsening acne during summer, while another cross-sectional study of 171 patients found that nearly half exhibited seasonal symptom variation, with 40% experiencing aggravation in warmer months (25, 60–63). Chronic occupational or recreational outdoor exposure further increases acne prevalence, particularly when combined with pollution, traffic-related emissions, or industrial exposure, suggesting synergistic oxidative and inflammatory stressors (64).

#### 2.2.2 Ultraviolet radiation

UVR exerts complex, wavelength-dependent effects on acne. Both UVA and UVB induce sebaceous gland hyperplasia, stratum corneum thickening, increased sebum secretion, and comedogenesis (62, 63, 65, 66). UVR acts on keratinocytes, sebocytes, and immune cells to trigger the release of antimicrobial peptides (AMPs) and to activate innate immune pathways, while suppressing adaptive immunity. This immune shift alters the skin microbiome, facilitating *C. acnes* overcolonization UV-induced microbial damage generates pathogen-associated molecular patterns (PAMPs), interfering with UVR-induced immunosuppression, further amplifying local inflammation and acne flares (67, 68).

The clinical response to sunlight is bidirectional. Moderate exposure may transiently improve inflammatory lesions, whereas excessive or chronic exposure typically worsens disease severity (17). Long-wavelength UVA1 (340–400 nm), visible light (VL) (~400 nm), and infrared radiation exhibit anti-inflammatory and antimicrobial properties, reducing *C. acnes* colonization without increasing sebum production or proinflammatory cytokines (69). These properties underpin their use in selected phototherapy protocols for acne (70–74). The effects of UVB radiation are dose- and exposure-dependent. While low or intermittent UVB exposure may exert immunosuppressive and antimicrobial effects, higher or cumulative doses promote keratinocyte hyperproliferation, increased sebum production, and pro-inflammatory cytokine release, resulting in acne exacerbation (63, 75).

*C. acnes* produces endogenous porphyrins that are highly photosensitive. UV and VL excitation of these porphyrins generates cytotoxic ROS, contributing to bacterial killing and forming the basis for photodynamic and blue-light acne therapies (76, 77). Interestingly, a large retrospective cohort of 19,939 Chinese college students reported a protective association with low-dose exposure to UVB, while identifying UVA as a potential acne risk factor, underscoring population-specific and environmental modifiers (25, 75). Overall, UVR constitutes a complex physical exposome factor in acne. While short-term exposure may transiently suppress inflammation, cumulative or repeated exposure contributes to oxidative stress, dysregulated lipogenesis, altered lesion distribution, and post-inflammatory sequelae (25).

UV-induced ROS drive lipid peroxidation and DNA damage in sebocytes and keratinocytes, lowering the activation threshold of innate immune pathways. Redox imbalance activates inflammasome signaling, particularly the NLRP3 inflammasome, a key mediator of acne-associated inflammation (78, 79). Pharmacological redox modulation can attenuate inflammasome activation. Notably, auranofin, an orally administered anti-rheumatic gold compound that inhibits thioredoxin reductase, reduces oxidative stress and suppresses NLRP3 inflammasome activation under oxidative stress conditions, which highlights its potential relevance in redox-driven inflammatory skin disease such as acne (80–82).

However, not all studies confirm a direct association between UVR and acne. A cross-sectional study found no statistically significant relationship between UV exposure and acne severity, nor associations with family history, diet, or age at menarche (83).

#### 2.2.3 Ultraviolet radiation and skin of color

In individuals with skin of color (SOC), typically corresponding to Fitzpatrick skin phototypes IV-VI, the interaction between UVR, VL, and acne inflammation carries distinct clinical implications. While some SOC patients may experience transient improvement in inflammatory lesions during summer, others develop flares accompanied by post-inflammatory hyperpigmentation (PIH) or post-inflammatory erythema, particularly following excoriated or persistent inflammatory lesions (84, 85).

VL and UVA1 play a critical role in PIH pathogenesis in SOC. VL activates Opsin-3 photoreceptors in phototypes III–V, triggering Ca^2+^ influx and MITF (microphthalmia-associated transcription factor) -dependent melanogenesis, which regulates the expression of tyrosinase and other pigment-related genes, leading to redistribution of melanin toward the upper epidermis (86). VL and UVA may act synergistically, amplifying pigmentary sequelae even in the absence of severe inflammation.

Photosensitizing acne treatments further complicate management in SOC. Isotretinoin frequently necessitates dose reduction or interruption due to photosensitivity (87, 88). Tetracyclines, particularly doxycycline, are associated with significant phototoxicity: in a prospective study of 106 patients treated over two years, phototoxic reactions occurred in 20% receiving 150 mg/day and 40% receiving 200 mg/day, with a higher incidence in regions of intense solar exposure (89).

Finally, the widespread use of skin-lightening products in SOC populations poses additional risks. Female SOC users report high rates of adverse effects, including acne, rash, and melasma (90, 91). Mercury-containing skin-whitening creams, still used globally, act as melanotoxins and can cause severe nodulocystic acne, along with renal and neurological toxicity following chronic exposure (92). Health education and stricter regulation of hazardous cosmetics are therefore critical components of acne prevention and management in SOC.

#### 2.2.4 Sunbeds, photoprotection, and pigmentary sequelae

Despite well-documented risks, including photocarcinogenesis, photoaging, and PIH, some acne patients use sunbeds as self-treatment. Dermatologists should actively discourage this practice, particularly in patients receiving isotretinoin (93, 94).

Daily photoprotection is essential for all acne patients, and particularly critical for SOC due to PIH vulnerability (60, 95–97). Broad-spectrum sunscreens protect against barrier damage caused by UVR and pollution, thus preserving epidermal barrier integrity, reducing transepidermal water loss (TEWL), and providing anti-inflammatory, antioxidant, sebum-regulating, and antipollution benefits (5, 98). However, cosmetic acceptability remains a key determinant of adherence; lightweight, non-greasy, matte formulations designed for oily skin improve compliance (99–101).

Protection against UVA1 and VL is best achieved with mineral filters such as titanium dioxide and zinc oxide, particularly when combined with iron oxides that are superior at blocking VL. Because UVA1 and VL contribute to PIH, sunscreens containing mineral UV filters and pigmentary iron oxides are strongly recommended (102, 103), especially for SOC patients who are more prone to pigmentary sequelae (104–107). In a cross-sectional study (n = 208), acne clearance was the primary concern for light-skinned women, whereas PIH resolution was the top priority for over 40% of dark-skinned women, compared with only 8% of light-skinned participants (108).

Tinted sunscreens with iron oxides provide effective protection against UVA1 and VL while offering cosmetic camouflage, a rapid and inexpensive intervention shown to improve quality of life (109, 110). Rigorous photoprotection is also mandatory following chemical peels and laser procedures for acne (111).

### 2.3 Occupational and lifestyle factors

Non-inflammatory acne, primarily characterized by comedonal lesions, may result from occupational or environmental exposures that induce follicular occlusion without significant inflammation. Several occupational settings and environmental agents are recognized for their potential to cause acne. In a Greek cohort, acne prevalence was significantly higher among bitumen workers, footwear artisans, and paint industry employees, demonstrating the established entities of coal tar acne and oil acne (112–114). These conditions are attributed to chronic exposure to tar derivatives, cutting oils, and related lipophilic compounds that induce mechanical obstruction of sebaceous ducts and promote comedone formation. Males appear more frequently affected, likely due to both higher occupational exposure and a greater baseline predisposition to acne (113, 114). Although such exposures are less common today, it is suggested that insoluble organic compounds such as coal tar and crude oil interact with keratinous material within follicles, forming obstructive plugs (113).

#### 2.3.1 Chloracne and related environmental acneiform eruptions

Chloracne is an acne variant caused by environmental pollutants and represents a chronic occupational acneiform dermatosis. It is caused by halogenated aromatic hydrocarbons like polychlorinated dibenzo-p-dioxins and polychlorinated biphenyls (PCBs) and typically manifests weeks to months after exposure (115). Histopathologically, it features keratinocyte hyperplasia, follicular hyperkeratosis, and cyst formation in the absence of significant inflammation (116).

A documented case of chloracne following accidental PCB leakage demonstrated clinical resolution with appropriate management, emphasizing the reversible nature of the condition when exposure is eliminated (117). Similarly, acneiform eruptions associated with polycyclic aromatic hydrocarbons (PAHs) from tobacco smoke suggest overlapping pathogenetic mechanisms between environmental pollutants and occupational acne (118).

#### 2.3.2 Healthcare workers and PPE-related acne

During the COVID-19 pandemic, acne associated with prolonged face mask use (“maskne”) emerged as a common occupational skin condition among healthcare workers (119, 120). Acne vulgaris and seborrheic dermatitis were among the most frequently reported conditions linked to personal protective equipment (PPE) use (121–125). Several observational studies in healthcare settings demonstrated a clear association between prolonged mask use and either new-onset acne or exacerbation of pre-existing acne, supporting the concept of PPE-related acne as a distinct occupational entity (120).

The pathogenesis involves the creation of a warm, humid microenvironment under face masks, which enhances sebum production and follicular occlusion (126). Mask-related factors, including material type, tight fit, duration of daily use, and repeated or prolonged reuse, have been consistently identified as contributors to acne risk among healthcare workers and other mask-wearing populations (122, 127, 128). In addition, friction and pressure at mask contact sites may induce mechanical acne, particularly over the cheeks, chin, and nasal bridge (129).

In addition, increased anxiety levels and psychological occupational stress among healthcare professionals during the pandemic may have exacerbated acne symptoms independently of mechanical or occlusive factors (130–132).

#### 2.3.3 Smoking and acne vulgaris

The relationship between smoking and acne vulgaris remains controversial. Some investigations have indicated a protective role of smoking, including an online survey reporting a 30% lower acne prevalence among smokers (133), and others confirming an inverse association (134, 135). Conversely, several studies identified smoking as an aggravating factor (136–138), while others found no significant relationship (139). A meta-analysis concluded that smoking is a risk factor for adult acne in Asian populations (140).

Postadolescent acne typically manifests as inflammatory lesions affecting the lower face and neck. However, a distinct comedonal postadolescent acne (CPAA) phenotype characterized by numerous micro- and macrocomedones with minimal inflammation, has been strongly linked to cigarette smoking and is now considered one of the most frequent types of smoking-associated adult acne (141).

Tobacco and cannabis consumption act as human-derived environmental pollutants capable of disrupting skin homeostasis and potentially acne pathogenesis (133, 138, 142). However, a definitive causal link between smoking and acne remains unproven. Cigarette smoke contains thousands of toxic compounds, including nicotine, carbon monoxide, tar, formaldehyde, hydrogen cyanide, ammonia, and heavy metals, which compromise skin integrity by increasing TEWL and promoting premature connective tissue damage (135). Smoking upregulates matrix metalloproteinases (MMP-1 and MMP-3), accelerates collagen and elastin degradation, suppresses collagen synthesis, impairs wound healing, and promotes elastosis, collectively weakening the pilosebaceous unit and facilitating acne development (143–145).

Mechanistic studies have showed that smoking induces acne pathogenesis through proinflammatory and oxidative pathways. Cigarette smoke increases interleukin-1α (IL-1α) expression, a key cytokine in comedogenesis, early acne lesion formation and follicular inflammation (135, 138, 141, 146). Meantime, smoking generates excessive ROS, leading to lipid peroxidation, including oxidized squalene accumulation, which amplifies inflammatory signaling and acne severity (147). These oxidative mechanisms closely resemble those triggered by air pollutants such as PM_2.5_ and ozone, reinforcing the concept of a convergent oxidative acne exposome.

Additionally, cigarette smoke alters sebocyte lipid metabolism by downregulating scavenger receptor class B type 1 (SR-B1), a key regulator of sebaceous lipid uptake and homeostasis. Reduced SR-B1 expression results in qualitative sebum alterations, promoting follicular occlusion, microbial dysbiosis, and inflammation (148, 149).

#### 2.3.4 Cannabis and cannabinoids in acne vulgaris

The global use of medical cannabis products (MCPs), containing tetrahydrocannabinol (THC) and/or cannabidiol (CBD), has expanded although dermatologic evidence remains limited (150). Cannabinoids exert anti-inflammatory, antipruritic, anti-aging, and antineoplastic effects through interaction with the cutaneous endocannabinoid system (ECS), which regulates keratinocyte activity, immune balance, and sebaceous gland function (150, 151).

Cannabidiol (CBD), the major non-psychoactive phytocannabinoid, exhibits sebostatic, antiproliferative, and anti-inflammatory activity. In human sebocytes and skin organ culture, CBD inhibited lipogenesis induced by arachidonic acid, linoleic acid, and testosterone, reduced proliferation via transient receptor potential vanilloid-4 (TRPV4) activation, and downregulated nuclear receptor interacting protein-1 (NRIP1) through inhibition of the ERK1/2 MAPK pathway. CBD also upregulated tribbles homolog 3 (TRIB3) via A_2_A adenosine receptor signaling, suppressing NF-κB–mediated inflammation (152). Comparative analyses revealed that cannabichromene (CBC) and THC exert sebostatic effects, whereas cannabigerol (CBG) and cannabigerovarin (CBGV) show pro-sebogenic properties (153). In a split-face clinical trial, a 3% cannabis seed extract cream applied twice daily for 12 weeks significantly reduced sebum production and erythema without adverse effects (154).

Hemp seed hexane extract (HSHE) has also shown antimicrobial and anti-inflammatory activity against *C. acnes*, downregulating iNOS, COX-2, IL-1β, and IL-8 expression, and suppressing NF-κB, MAPK, ERK, and JNK activation. HSHE further inhibited 5-lipoxygenase and MMP-9, promoted collagen synthesis, and modulated AMPK and AKT/FoxO1 pathways, further reducing inflammation and lipogenesis (155).

Despite increasing consumer use of cannabis-based skincare products, often without dermatologic supervision, epidemiologic data remain inconsistent. In a survey of 504 adults, 17.6% reported using over-the-counter cannabis formulations, primarily for acne (28.4%) and psoriasis (26.1%), often without dermatologic guidance (150). A French survey (>10,000 participants) associated regular cannabis use with higher acne prevalence (OR = 2.88; 95% CI: 1.55–5.37) (142), whereas subsequent international studies failed to confirm this, likely due to self-reporting bias and legal constraints (23, 64, 156).

#### 2.3.5 Cosmetics and facial practices as chronic exposomal stressors in acne vulgaris

Cosmetic products constitute chronic, repetitive, low-dose exposomal exposures applied directly to acne-prone skin and have been increasingly implicated in acne exacerbation. Historically described as acne cosmetica, their acneigenic potential is primarily mediated through comedogenicity, defined as the induction of microcomedone formation within the follicular infundibulum, the earliest lesion of acne vulgaris (157). Comedogenic ingredients such as long-chain fatty acids, fatty alcohols, esters, lanolin derivatives, waxes, and occlusive oils, can promote microcomedone formation by altering follicular keratinization and sebum composition (158).

From an exposomal perspective, cosmetic-induced occlusion alters the biophysical and biochemical microenvironment of the follicle by reducing oxygen tension and modifying lipid composition, which favors early microcomedone formation (23, 159, 160). The acneigenic risk is not solely dependent on individual ingredients but also on formulation complexity, frequency of application, duration of contact, and cumulative exposure, reinforcing the exposome concept of dose and chronicity (23).

Acne vulgaris frequently persists into adulthood, particularly in women, with prevalence estimates of 12% in women and 3% in men over 25 years of age (161–164). Notably, 40% of adult patients report acne aggravation following cosmetic use, especially with skin-lightening products, underscoring cosmetics as clinically relevant acne-modifying exposures in adult populations (165). Conversely, gender of male, warm water for washing face, frequent sun protection, frequent use of moisturizing products and extended use of pad and phone were identified as independent risk factors for acne, while physical exercise and cosmetic usage were identified as independent protective factors. Moreover, participants who utilized a higher number of moisturizing products were associated with an increased risk of developing acne (166).

The epidermal barrier, composed primarily of ceramides, cholesterol and free fatty acids within the stratum corneum, regulates TEWL and protects against microbial and chemical insults (167, 168). Skin surface pH is a critical determinant of barrier integrity and microbial homeostasis, and deviations from physiological acidity predispose to inflammatory dermatoses, including acne vulgaris (19, 169, 170). Modern cleansers for acne-prone skin are therefore formulated with acidic syndets and buffered with organic acids or salts (e.g., lactic or citric acid, sodium lactate or citrate) and frequently incorporate hydroxy acids (salicylic, glycolic, lactic, mandelic), as well as ascorbic, ferulic, and linoleic acids, to support keratinization control and barrier function (171). In contrast, many traditional soaps and shampoos remain alkaline (pH 9–10 and 6–7, respectively), a profile considered barrier-disruptive and suboptimal for acne-prone skin (172).

Behavioral facial practices constitute an important exposomal factor, besides the product composition. Layering multiple cosmetic products, prolonged or overnight wear, and inadequate removal increase contact time and increase acne risk (23, 173). Additionally, mechanical and occlusive practices, including mask use, repetitive face touching, pressure from devices, or friction from accessories, contribute to acne mechanica, characterized by follicular injury, sweat retention, and localized inflammation (157). Case-control studies confirm increased odds of acne associated with powders, foundations, and cleansers containing comedogenic formulations, independent of hormonal or familial confounders (173). Conversely, data on modern non-comedogenic and barrier-supportive formulations are not inherently acneigenic, emphasizing the pivotal role of ingredient selection, formulation science, and exposure behavior (174–177).

#### 2.3.6 Cosmeceuticals in acne and skin of color

Cosmeceuticals and dermocosmetics contain biologically active ingredients targeting key acne pathways, including sebogenesis, inflammation, follicular hyperkeratinization, oxidative stress, and epidermal barrier dysfunction. Growing evidence supports their use as adjunctive agents in mild acne, acne-prone skin, and maintenance therapy, improving lesion counts, tolerability, and patient-reported outcomes (178–181). Active ingredients such as niacinamide, azelaic acid, salicylic acid, antioxidants, and selected plant-derived extracts (*Silybum marianum, Myrtus communis, Cirsium eriophorum*) demonstrate anti-inflammatory, sebum-modulating, and barrier-supportive effects when appropriately formulated (180–182). Mechanistically, these bioactive ingredients work through distinct pathways. Salicylic acid induces desquamation *via* corneodesmosome disruption and cyclooxygenase inhibition (183, 184). Concurrently, azelaic acid inhibits *C. acnes*, reduces keratinocyte hyperproliferation and mitochondrial oxidoreductase activity (185, 186). Niacinamide complements these actions by enhancing ceramide synthesis, strengthens barrier integrity and inhibits pro-inflammatory cytokine release (187, 188). Barrier-repair cosmeceuticals containing ceramides, glycerin, and panthenol improve tolerability of topical retinoids and benzoyl peroxide and enhance adherence (178, 189).

Patients with SOC exhibit increased susceptibility to post-inflammatory hyperpigmentation, keloidal scarring, and irritant dermatitis. In this population, cosmeceuticals may mitigate acne-associated dyspigmentation through inflammation control and gentle pigment modulation; however, inappropriate use of comedogenic, fragranced, or irritating formulations may exacerbate both acne and hyperpigmentation (174, 190, 191). Aggressive or unregulated depigmenting products, frequently marketed to skin of color populations, may further induce irritant or contact dermatitis, worsening outcomes (179, 192).

Taken together, cosmetics and cosmeceuticals represent important, yet modifiable components of the acne exposome. When appropriately formulated and used, cosmeceuticals serve as valuable adjuncts in mild acne, maintenance therapy, and supportive care alongside pharmacological treatments. However, they should not replace evidence-based medical therapies in moderate-to-severe disease, as their effects remain modest and misuse may delay effective intervention (179, 193–195). Dermatologist-guided selection and patient education remain essential to optimize benefit and minimize harm across diverse skin types (178, 192, 195, 196).

## 3 Nutrition and acne

Acne has often been described as a “Western disease” due to its high prevalence in Western societies, where lifestyle and dietary habits significantly differ from those in non-Western populations (197). The strong correlation between diet and acne suggests that certain dietary patterns, particularly those common in Western diets, may exacerbate acne by affecting insulin sensitivity and hormone levels (198). Mechanistic and clinical data consistently implicate Western dietary components - particularly dairy products, high-glycemic-load foods, and specific protein and fat sources - in acne pathophysiology through insulin/IGF-1–mTORC1–FoxO1–driven pathways (199–202).

### 3.1 Dairy products

Cow’s milk consumption has been consistently linked to the development of acne, with skim milk showing a stronger association than whole milk (139). This is attributed to milk-derived bioactive molecules, including insulin-like growth factor-1 (IGF-1) and other hormones that can trigger acne by activating the FoxO1 and mTORC1 pathways (203–205). Nutritional and metabolic exposome factors influence insulin/IGF-1–mTORC1 signaling, androgen bioavailability, oxidative stress, and innate immune activation (206). Collectively, these pathways promote sebocyte lipogenesis, follicular hyperkeratinization, and inflammasome-mediated IL-1β release, leading to microcomedone formation and sustaining inflammatory lesion development (207). Meta-analyses support a dose-dependent relationship between milk intake and increased acne risk, though the impact of different forms of dairy remains debated (201, 208–210).

### 3.2 High-glycemic load diets

Diets high in glycemic load have been strongly associated with acne (198). High-glycemic foods cause rapid spikes in blood sugar levels, leading to increased insulin production and subsequent activation of sebaceous glands. Clinical studies have shown that individuals who adopt a low-glycemic-load diet experience significant improvements in acne symptoms, along with enhanced insulin sensitivity and weight loss (211, 212). These clinical effects are supported by mechanistic evidence linking glycemic load to mTORC1 overactivation, androgen bioavailability, and inflammatory signaling within the pilosebaceous unit (199–202).

### 3.3 Omega-3 fatty acids and polyunsaturated fatty acids

Omega-3 polyunsaturated fatty acids (PUFAs), particularly eicosapentaenoic acid (EPA) and docosahexaenoic acid (DHA) derived from fish and marine sources, are consistently associated with reduced acne incidence and severity, in contrast to Western diets rich in saturated fats and omega-6 PUFAs that increase acne risk (1, 139, 198). Lipid profiling studies demonstrate that acne patients exhibit significantly lower circulating and erythrocyte EPA levels, indicating a systemic pro-inflammatory lipid profile (213–215).

Omega-3 fatty acids exert pleiotropic anti-acne effects by attenuating IGF-1 signaling, suppressing mTORC1 activation, and inhibiting TLR-1/2–mediated inflammatory pathways and NLRP3 inflammasome activation within sebocytes and innate immune cells (216). They also shift eicosanoid production toward less inflammatory and pro-resolving lipid mediators, counterbalancing arachidonic acid–driven inflammation (217).

Randomized controlled trials demonstrate that oral omega-3 supplementation, alone or combined with gamma-linolenic acid, significantly reduces inflammatory lesion counts and cytokines such as IL-8 (218–220). Omega-3 fatty acids modulate the gut–skin axis by increasing the relative abundance of short-chain fatty acid (SCFA)–producing taxa, including Lachnospiraceae and Ruminococcaceae, while reducing pro-inflammatory Proteobacteria (221, 222). This microbial shift enhances the production of anti-inflammatory metabolites such as butyrate and propionate, which exert systemic immunomodulatory and epigenetic effects on acne pathogenesis (223, 224). Omega-3 supplementation may also mitigate isotretinoin-associated adverse effects, including xerotic cheilitis and mucocutaneous dryness and hypertriglyceridemia in metabolically predisposed individuals (225, 226).

Experimental sebocyte models demonstrate that linoleic acid induces distinct transcriptional and lipidomic changes compared with saturated (palmitic acid) and omega-6 arachidonic acid exposure, underscoring fatty acid–specific effects on sebocyte inflammation and differentiation (227). Genetic and MR analyses support a causal link between circulating PUFA profiles, especially omega-3 levels, and acne susceptibility, providing population-level evidence that lipid metabolism is a contributory factor in acne pathogenesis (228, 229).

### 3.4 Chocolate and whey protein

The relationship between chocolate consumption and acne remains controversial. While some studies found no significant correlation (139, 216, 230), others reported an association between chocolate intake and acne exacerbation. In an online survey, chocolate consumption increased acne likelihood by up to 30% (133), and pure cocoa ingestion or daily dark chocolate intake triggered flare-ups in acne-prone males (231, 232). Proposed mechanisms include sugar- and leucine-driven activation of insulin/IGF-1–mTORC1 signaling, which may outweigh cocoa’s intrinsic antioxidant properties (199, 201).

Whey protein supplementation has similarly been implicated in acne due to its high leucine content and potent stimulation of IGF-1 and mTORC1 pathways. Case reports describe acne flares in athletes using whey protein supplements (233–235). However, recent randomized controlled trials have not consistently demonstrated significant changes in acne lesion counts with whey protein use over six months (233, 236, 237). Thus, chocolate and whey protein may exacerbate acne in susceptible individuals, though interindividual variability and dietary context remain critical (199, 201).

## 4 Sebaceous gland as an exposome sensor

### 4.1 Sebocyte intrinsic stress responses triggered by the exposome

Sebocytes are highly responsive stress-sensing cells that translate environmental and internal exposome signals into inflammatory, metabolic, and cell fate decisions (238, 239). Exposure to physical, chemical, microbial, and metabolic stressors activates conserved danger-sensing pathways - including inflammasome signaling, autophagy, and regulated cell death - placing intrinsic sebocyte stress responses at the center of acne initiation, amplification, and chronicity (240, 241). Within this context, hypoxia represents a fundamental metabolic stressor of the pilosebaceous unit. The pilosebaceous unit is intrinsically prone to hypoxia due to follicular occlusion, sebum accumulation, microbial overgrowth, and inflammatory edema, with external factors such as occlusive clothing, mechanical pressure, heat, and impaired microcirculation further aggravating hypoxic conditions (242). Hypoxia induces sebaceous lipogenesis *via* hypoxia-inducible factor (HIF)–dependent pathways, promoting lipid accumulation and creating an anaerobic microenvironment favouring proliferation of *C. acnes* (243, 244).

### 4.2 Inflammasome activation and ion channels

Inflammasome activation represents a key molecular link between exposomal stress and sebocyte-driven inflammation. The NLRP3 inflammasome, activated by oxidative stress, mitochondrial dysfunction, and microbial signals, has emerged as a critical mediator of acne-associated inflammation. 5-Aminolevulinic acid photodynamic therapy (ALA-PDT), a treatment widely used in dermatology for inflammatory and neoplastic skin conditions, induces IL-1β secretion in human SZ95 sebocytes *via* NLRP3 inflammasome activation (245), demonstrating that oxidative and phototoxic stress can directly trigger innate immune signaling within sebocytes independent of immune cell infiltration. Given the ubiquity of UV- and photo-oxidative stress, these mechanisms are likely relevant beyond therapeutic settings.

Ion channel–mediated stress sensing has been implicated in sebocyte inflammation. TRPV3, a temperature- and chemical-sensitive cation channel, promotes sebocyte inflammation through transcriptional modulation of TLR2 (246). As TRPV3 can be activated by thermal stress and chemical irritants, this pathway provides a direct molecular link between environmental exposure and inflammatory signaling within the pilosebaceous unit.

### 4.3 Autophagy, ferroptosis, and sebocyte cell fate

Beyond inflammatory signaling, exposome-induced stress influences sebocyte cell fate through modulation of autophagy and regulated cell death pathways. Autophagy is a key homeostatic mechanism controlling lipid metabolism, organelle turnover, and stress adaptation in sebocytes. Clinical and experimental evidence indicates that autophagy activation improves skin barrier function and alleviates acne symptoms (247). Mechanistically, autophagy regulates sebaceous lipid production and contributes to the sebosuppressive effects of retinoic acid, one of the most effective systemic acne therapies, suggesting that retinoids may partially restore impaired stress-adaptive autophagic flux in acne-prone sebocytes (248).

Ferroptosis, an iron-dependent form of lipid peroxidation-driven cell death, has recently been implicated in sebaceous gland targeting strategies. Nanoparticle-based photothermal therapy induces sebaceous gland ferroptosis, leading to reduced sebaceous activity and clinical acne improvement (249).

## 5 Intrinsic exposome

### 5.1 Genetic susceptibility in acne vulgaris

The contribution of genetic factors to acne vulgaris is substantial and well supported by epidemiologic, familial, and genome-wide association studies (GWAS) (**Table 1**). Acne is now regarded as a complex polygenic disorder, with multiple susceptibility loci influencing sebaceous gland activity, androgen signaling, inflammation, follicular keratinization, and innate immunity. Comprehensive reviews of the genetic architecture of acne have identified numerous risk variants across diverse populations, confirming the strong heritable basis of the disease (250–252).

**Table 1:** Genetic determinants and related pathways implicated in acne vulgaris.

Study type	Gene/locus	Biological pathway	Associated clinical phenotype
Twin studies	Multiple loci	Heritability 81-85% environment 15-19%	Acne development
GWAS	1q41, 5q11.2, 11q13.1	TGF−β signaling	Associated with severe acne
	1q24.2, 11p11.2	Follicular development	Risk to severe acne
GWAS meta−analysis	Wnt, TGF−β, ECM,	Hair follicle development, morphogenesis	Risk to severe acne
	TNF (rs1800629)	Inflammatory signaling	Reduced risk of mild acne (A allele)
	CYP17A1 (rs743572)	Androgen biosynthesis	Reduced risk of severe acne (protective allele)
	FST (rs629725)	Follicular development, inflammation (TGF-β)	Modestly increased risk of acne (A allele)
	TLR4 (3′-UTR SNPs)	Inflammatory signaling	Acne susceptibility
Mendelian randomization	Proteome → Genetic drug targets	Immune modulation, lipid metabolism, and sebocyte function	Acne treatment
	Metabolite-linked loci	Systemic serum metabolites and inflammation	Acne risk

Familial aggregation provides early evidence for genetic predisposition. A meta-analysis showed that individuals with affected first-degree relatives have more than a threefold increased risk of developing acne (OR 3.41; 95% CI 2.31–5.05) (139). Similarly, in a Chinese Han population, first-degree relatives of acne patients were 4.05 times more likely to develop the disease compared to relatives of healthy controls (253). In Lithuanian adolescents, family history ranked among the strongest predictors of acne, particularly when both parents were affected (OR 2.6), alongside metabolic and pubertal factors such as BMI >25, menarche in girls, and facial hair growth in boys (254).

Twin studies further quantify the genetic contribution. In female twin cohorts, additive genetic effects accounted for 81% (95% CI 73–87%) of acne variance, with environmental factors explaining the remaining 19% (255). Similar estimates were reported in large Australian twin studies, attributing up to 85% of acne susceptibility to genetic influences (256). These findings position acne among dermatologic diseases with one of the highest heritability estimates.

Large-scale GWAS have refined the genetic architecture of acne, identified multiple reproducible risk loci and reinforcing its polygenic nature. Genome-wide significant loci replicated across populations include 1q41, 5q11.2, and 11q13.1, regions enriched for genes involved in TGF-β signaling, as well as additional loci at 1q24.2 and 11p11.2 (257–259). Subsequent meta-analyses expanded the catalog of susceptibility loci, implicating pathways related to sebaceous gland biology, immune regulation, epidermal barrier function, and follicular development (260). A landmark GWAS meta-analysis demonstrated enrichment of acne risk loci in pathways governing hair follicle morphogenesis, extracellular matrix remodeling, and 249/TGF-β signaling, demonstrating the pilosebaceous unit as a genetically primed target organ (261). Aberrant activation of TGF-β and Wnt/β-catenin signaling contributes to acne scar formation through dysregulated fibroblast activity and extracellular matrix remodeling, while crosstalk between these pathways and retinoid signaling modulates epidermal differentiation, collagen turnover, and interindividual variability in therapeutic response (262–266).

Candidate gene studies and meta-analyses further support these associations. A systematic review encompassing 51 studies across Asian and Caucasian populations identified 60 acne-associated genes/loci with over 100 variants, many located in regulatory or coding regions (267). Frequently studied genes included TNF, interleukins, and cytochrome P450 family members. While many variants showed modest or nonsignificant pooled effects, specific alleles demonstrated population-level associations: the TNF rs1800629 A allele was associated with reduced risk of mild acne (pOR 0.60; 95% CI 0.33–0.86), the CYP17A1 rs743572 T allele with lower severe acne risk (pOR 0.59; 95% CI 0.40–0.79), and the FST rs629725 A allele with a modestly increased risk (pOR 1.19; 95% CI 1.14–1.23). Novel single-nucleotide polymorphisms (SNPs) in the 3′ untranslated region of TLR4 further implicate innate immune sensing in acne susceptibility (267).

Beyond risk prediction, Mendelian randomization (MR) approaches have begun to translate genetic findings into causal inference and therapeutic prioritization. Proteome-wide MR analyses identified genetically supported protein targets involved in immune modulation, lipid metabolism, and sebocyte function, highlighting potential novel druggable pathways for acne therapy (268). In parallel, MR analyses investigating circulating metabolites demonstrated causal relationships between specific serum metabolic profiles and acne risk, supporting a genetically mediated link between systemic metabolism, inflammation, and acne pathophysiology (269). Together, these data position acne genetics at the intersection of developmental biology, immunometabolism, and precision therapeutics.

Gene expression and pathway analyses provide functional validation of genetic risk. Inflammatory acne lesions exhibit increased expression of matrix metalloproteinases (MMP-1, MMP-3), IL-8, and antimicrobial peptides including human β-defensin-4 and granzyme B (270). Immunohistochemical studies confirm elevated β-defensin-2 expression in lesional and perilesional skin, with more modest changes in β-defensin-1 (271), reflecting enhanced innate immune activation and tissue remodeling. Although genetic susceptibility clearly influences acne risk, its relationship with disease severity is less consistent, suggesting that downstream regulatory mechanisms and environmental modifiers shape the clinical phenotype (1). Genetic risk establishes a primed pilosebaceous unit upon which exposomal and epigenetic modifiers act.

### 5.2 Epigenetic and post-transcriptional regulation in acne vulgaris

Epigenetic mechanisms constitute a critical interface between genetic susceptibility and environmental exposures in acne vulgaris, enabling sustained activation of inflammatory, metabolic, and immune pathways without alterations in DNA sequence (**Table 2**). Through histone modifications, non-coding RNAs, and DNA methylation changes, epigenetic regulation shapes sebocyte and keratinocyte responses to microbial, hormonal, metabolic, and environmental stressors, contributing to acne initiation, severity, phenotypic heterogeneity, and chronicity. Importantly, sebocytes function as highly plastic, epigenetically regulated endocrine–immune units rather than passive lipid-producing cells (239, 240, 272–274). Epigenetic dysregulation may also explain the mechanistic links between acne vulgaris, acne syndromes, mosaicism, and exposome-driven interindividual variability (275).

**Table 2:** Key epigenetic mechanisms implicated in acne vulgaris.

Epigenetic mechanism	Key molecules	Sample	Molecular alterations	Clinical outcome
Histone acetylation	HDAC1 ↑	Serum, Keratinocytes	Pro−inflammatory transcriptional bias	Acne severity
	SCFAs (butyrate) by *C. acnes*	Sebocytes, Keratinocytes	HDAC inhibition	Chronic inflammatory dermatoses/acne vulgaris
miRNAs	miR−146a−5p ↑, miR−143 ↑	Acne lesions	Suppression of TNF, IL-6, and IL-8 (anti−inflammatory role)	Limits skin inflammation
	miR-146a	Sebocytes (SZ95) & acne lesions	Regulates TLR1/2- and TLR4-mediated inflammation, proliferation, and lipid synthesis through GNG7	Acne inflammation and lipid production
	miR−21, miR−155	Acne lesions	Sustained cytokine release (pro−inflammatory role)	Severe acne symptoms
	miR−21, miR−150	Plasma	Post-inflammatory remodeling and fibrosis	Acne severity with scarring
lncRNA	H19 ↑ by *C. acnes*	Keratinocytes	Activation of miR-196a/TLR2/NF−κB axis	Inflammation within the pilosebaceous unit
circRNAs	circ_0105040	Keratinocytes	Sponges miR−146a	*C. acnes* biofilm-induced inflammation
DNA methylation	DMEGs (PTPRC, CD86, ITGAM, CD8A, IL1B, TNF, TLR4, ITGB2, SPI1, LCP2, and STAT3)	Lymphocytes, Myeloid cells	Enhanced immune activation	Early-stage inflammatory acne lesions
	ZDHHC20	Acne lesions	Reduced protein palmitoylation and immune signaling	Reduced acne vulgaris risk

#### 5.2.1 Histone modifications and chromatin dynamics

**Figure 2:**
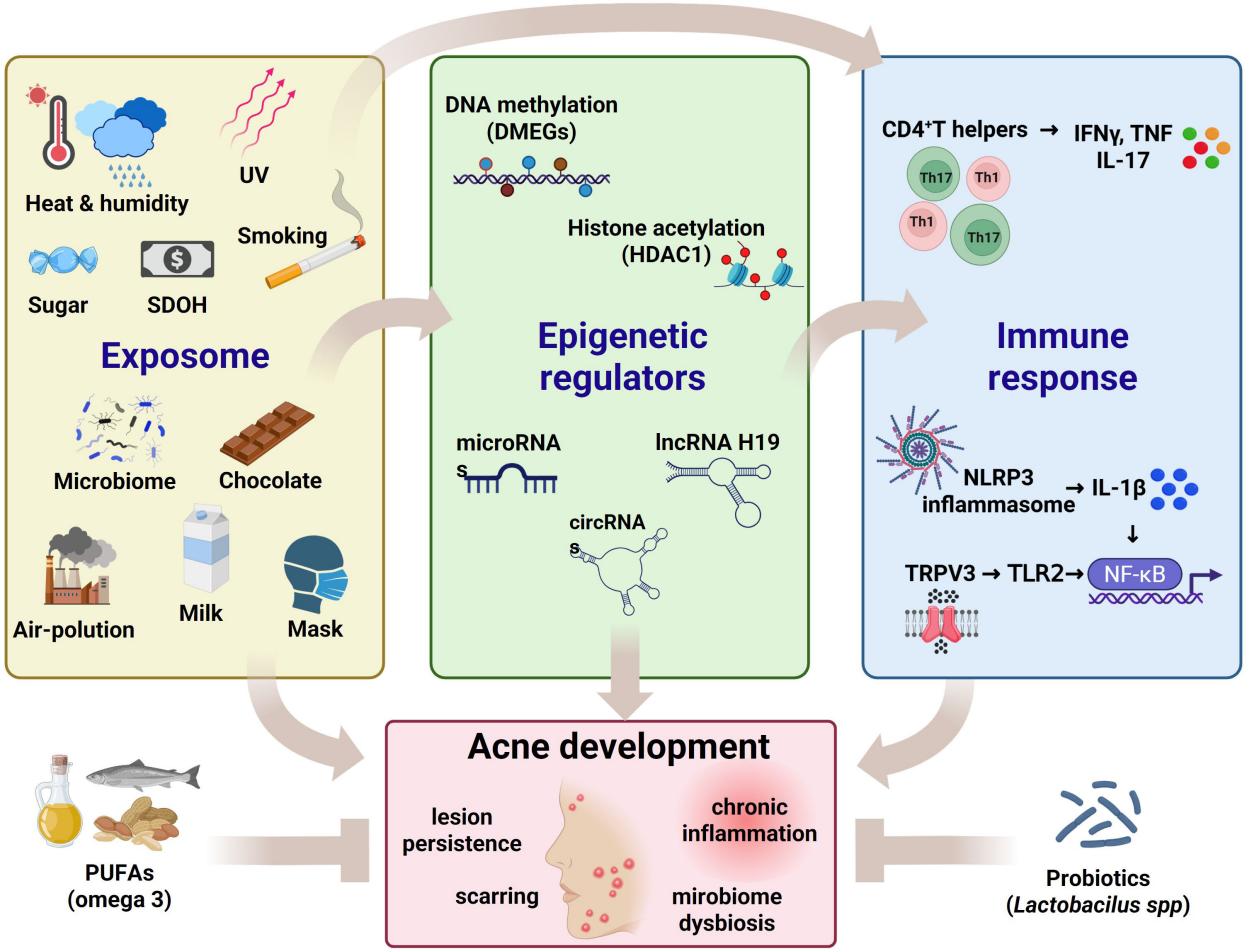
Exposome–epigenetic–immune crosstalk in acne vulgaris. Cumulative exposomal factors, including climate-related stressors (heat, humidity, UV), lifestyle and dietary components (high sugar intake, milk, chocolate), smoking, air pollution, microbiome perturbations, and social determinants of health (SDOH), shape acne susceptibility. These extrinsic and intrinsic factors converge on epigenetic regulators, inducing DNA methylation changes (differentially methylated genes, DMEGs), altered histone acetylation (e.g., HDAC1 activity), and modulation of non-coding RNAs (microRNAs, lncRNAs such as H19, and circRNAs). Epigenetic reprogramming influences innate and adaptive immune responses, promoting CD4^+^ T-helper polarization (Th1/Th17) with increased IFN-γ, TNF, and IL-17 production, activation of the NLRP3 inflammasome with IL-1β release, and TRPV3–TLR2–NF-κB signaling. The integrated effects of these pathways sustain cutaneous inflammation, microbiome dysbiosis, lesion persistence, and scarring, ultimately driving acne development and chronicity. On the other hand, probiotics and PUFAs have an anti-inflammatory effect.

Altered histone acetylation plays a central role in acne-associated inflammation and sebaceous gland hyperactivity. Elevated serum levels of histone deacetylase 1 (HDAC1) have been reported in acne patients, indicating a systemic epigenetic milieu favoring transcriptional repression of anti-inflammatory genes and amplification of pro-inflammatory signaling (276). HDAC-driven chromatin condensation influences cytokine expression, innate immune responses, and keratinocyte differentiation, correlating with disease severity. (**Figure 2**).

Pharmacological evidence further supports the functional relevance of histone regulation in acne. Minocycline suppresses sebocyte lipogenesis through inhibition of p300 histone acetyltransferase (HAT) activity in human SZ95 sebocytes, reducing acetylation at lipogenic gene promoters and downregulating lipid synthesis (277). These findings suggest that commonly used acne therapies may exert clinically meaningful epigenetic modulation on sebocyte metabolism, beyond antimicrobial activity.

Epigenetic control of sebocyte lipogenesis represents a core pathogenic axis. Key transcriptional regulators of lipid synthesis, including sterol regulatory element-binding protein-1 (SREBP-1) and peroxisome proliferator-activated receptor-γ (PPAR-γ), are tightly governed by chromatin accessibility and epigenetic co-regulators. *In vivo* and *in vitro* studies using synthetic decoy oligodeoxynucleotides targeting SREBP-1 and PPAR-γ demonstrated marked suppression of sebaceous lipogenesis and improvement of acne-like inflammation, confirming that sebocyte hyperactivity is epigenetically programmed rather than purely hormonally driven (278).

Metabolite-sensing G-protein–coupled receptors further link metabolic cues to epigenetic regulation. GPR119 has emerged as a regulator of sebocyte lipid synthesis, differentiation, and inflammatory signaling (279). By responding to lipid-derived metabolites, GPR119 likely integrates nutritional and metabolic exposome signals into epigenetically controlled transcriptional networks, reinforcing links between diet, metabolism, and acne.

Emerging evidence suggests that ion channels indirectly shape sebocyte immune and metabolic phenotypes via epigenetic mechanisms. The transient receptor potential melastatin 5 (TRPM5), a calcium-activated cation channel, has been implicated in sebocyte regulation; its antagonist triphenylphosphine oxide increases sebaceous lipogenesis and alters sebocyte immune profiles in a TRPM5-independent manner (280), implying downstream or off-target epigenetic effects on lipid and inflammatory gene expression. These findings highlight the complexity of sebocyte regulatory networks and support the concept of sebocytes as environmental sensors integrating ionic, microbial, and metabolic signals into epigenetically stabilized functional states.

#### 5.2.2 DNA methylation alterations and immune programming

Acne lesions display distinct DNA methylation profiles compared with normal skin, particularly affecting genes involved in innate and adaptive immunity. Genome-wide methylation analyses identified 31,134 differentially methylated sites across 22 chromosomes and 770 differentially methylated and expressed genes (DMEGs), which are defined as genes exhibiting concurrent changes in DNA methylation status and transcriptional expression. Eleven key DMEGs—*PTPRC, CD86, ITGAM, CD8A, IL1B, TNF, TLR4, ITGB2, SPI1, LCP2*, and *STAT3*—are central to immune activation and inflammatory signaling in early acne lesions (281). Notably, methylation changes are stage-dependent, with the most pronounced alterations observed in early inflammatory acne, implicating DNA methylation as a driver of lesion initiation rather than late persistence.

Systemic epigenetic reprogramming is supported by peripheral blood analyses showing altered immune cell composition (reduced NK cells, increased neutrophils) and methylation changes in loci such as *PDGFD*, *LOC105375130* (CARD11 signaling), and *ARHGEF10*, genes involved in NF-κB activation, cytoskeletal dynamics, migration, and apoptosis (282). Population-based studies further demonstrate DNA methylation–mediated genetic risk in young men with severe acne, indicating that epigenetic modifications amplify inherited susceptibility (283). Importantly, not all methylation changes are pathogenic; methylation at cg18095732 regulating *ZDHHC20* expression has been associated with reduced acne risk, highlighting protective epigenetic adaptations (284).

Epigenetic alterations converge on macrophage- and T-cell–mediated inflammation. Macrophages engulf *C. acnes* (285) a and activate the NLRP3 inflammasome, leading to IL-1β release, a key initiating event in acne pathogenesis (286–289). Both *IL1B* and *STAT3*, prominent DMEGs, participate in this axis, resembling inflammatory signaling observed in atherosclerosis (290). Notably, *C. acnes* directly activates the NLRP3 inflammasome in human sebocytes, reinforcing the sebaceous gland as an active immunological organ (78, 291).

Adaptive immunity further sustains inflammation. Acne lesions exhibit prominent infiltration of CD4^+^ Th1 and Th17 cells, correlating with lesion severity and chronicity (159, 292, 293). Epigenetically regulated genes such as *CD8A, PTPRC*, and *ITGB2* underscore adaptive immune recruitment and persistence (294), supporting contemporary models of acne as an immune-mediated disease characterized by dysregulated innate–adaptive crosstalk (286, 287). Finally, environmental and dietary exposures may modulate acne-associated epigenetic landscapes. Epigenetic dysregulation is increasingly recognized in inflammatory dermatoses, including rosacea (19) and hidradenitis suppurativa (HS), where methylation abnormalities affect genes involved in wound repair and keratinocyte proliferation (295–297). Given mechanistic overlaps between HS and acne (198, 298), these observations reinforce the centrality of the epigenome-inflammation axis. Dietary factors particularly milk-derived miRNAs, that survive pasteurization, may further influence acne epigenetics by targeting p53 and DNMT1, potentially attenuating tumor suppressor function and DNA methylation pathways (299). Persistent milk consumption may therefore represent a nutritional epigenetic trigger exacerbating acne and other Western inflammatory diseases linked to p53/DNMT1 dysregulation.

### 5.3 Non-coding RNAs in acne

Epigenetic control *via* microRNAs (miRNAs), long non-coding RNAs (lncRNAs), and circular RNAs (circRNAs) plays a crucial role in acne pathophysiology by regulating post-transcriptional gene expression in immune cells, keratinocytes, and sebocytes.

Several miRNAs involved in immune regulation, oxidative stress, and metabolic signaling have been implicated in acne. Both acne vulgaris and hidradenitis suppurativa (HS) share deregulated miRNA profiles primarily governing innate immune pathways. Anti-inflammatory miRNAs such as miR-146a-5p and miR-143 are upregulated upon *C. acnes* biofilm exposure, attenuating IL-6, IL-8, and TNF production through targeting of TLR2 and NF-κB signaling. Conversely, pro-inflammatory miRNAs, including miR-21 and miR-155, are overexpressed in acne lesions, promoting cytokine release and sustaining inflammation (300). Additionally, miR-146a connects TLR-mediated inflammatory responses with sebocyte proliferation and indirectly regulates lipid production via GNG7. Following TLR1/2 and TLR4 stimulation, miR-146a is upregulated in sebocytes and in acne lesions, where it suppresses cytokine secretion (IL-8) and chemotaxis, enhances proliferation, and reduces apoptosis. Simultaneous downregulation of GNG7 appears to shift sebocyte activity from proliferation toward increased lipid synthesis (301).

Oxidative stress–related miRNAs are particularly enriched in severe acne. Altered expression of miRNAs involved in redox balance and inflammatory amplification has been documented, reinforcing the link between oxidative stress and epigenetic dysregulation in acne severity (302). In line with this, dysregulated expression of FoxO1, mTOR-related miRNAs (miR-21, miR-29b, miR-98) has been associated with metabolic syndrome features in acne patients, suggesting convergence between metabolic signaling and epigenetic control (303).

Comparative profiling of acne patients with and without scarring identified 88 differentially expressed miRNAs, with miR-223, miR-21, and miR-150 emerging as key regulators. Plasma levels of miR-21 and miR-150 were significantly elevated in patients with scarring, implicating these miRNAs in post-inflammatory remodeling and fibrosis (304). More recently, miR-191-5p has been associated with acne severity and disease progression, highlighting its potential role as a prognostic biomarker (305). In addition, serum exosomal miRNA profiling revealed a marked downregulation of hsa-miR-124-3p in severe acne, implicating impaired immune resolution and T-cell regulation in advanced disease stages (306).

LncRNAs fine-tune inflammatory signaling within the pilosebaceous unit. The lncRNA H19 is induced in keratinocytes following *C. acnes* exposure and facilitates inflammation via the miR-196a/TLR2/NF-κB axis reinforcing innate immune activation within the pilosebaceous unit (307). Dysregulated lncRNA networks have been identified in familial acne inversa associated with *NCSTN* mutations, suggesting shared epigenetic regulatory principles across acneiform disorders (308).

CircRNAs add an additional regulatory layer by acting as miRNA sponges. Multiple circRNAs are downregulated in acne lesions, including circRNA_0084927, circRNA_0001073, circRNA_0005941, circRNA_0086376, and circRNA_001816, suggesting potential loss of post-transcriptional regulatory control (309). Functional studies have demonstrated that hsa_circ_0105040 promotes *C. acnes* biofilm–induced inflammation by sequestering miR-146a, thereby amplifying inflammatory signaling in human keratinocytes (310). These findings position circRNAs as critical modulators linking microbial stimuli to sustained inflammatory responses in acne (311).

## 6 Microbiome-epigenome crosstalk in acne vulgaris

The microbial exposome, consisting of skin - gut microbiota and their bioactive products, acts as a central mediator linking external and internal exposures to host epigenetic plasticity. Rather than reflecting simple microbial overgrowth, acne arises from qualitative shifts in microbial communities, strain-level functional activity and their metabolites, which reprogram gene expression in sebocytes, keratinocytes, and immune cells through epigenetic mechanisms (**Table 3**).

**Table 3:** Skin and gut microbiome alterations in acne vulgaris.

Microbiome site	Phylum/Species	Change in acne	Functional implication	Clinical correlation
Skin (Acne lesions)	*C. acnes/A1*	Increased	Chronic inflammatory lesions and persistence	Chronic acne
	Staphylococci	Increased	–	Acne severity
	*S. epidermidis*	Increased or decreased	Protective or competitive effects against *C. acnes*	Severity−dependent
	*Malassezia* spp.	Increased	Pro−inflammatory interaction	Inflammatory severity of acne
	Firmicutes *↑/*Proteobacteria *↓*	Increased ratio	Barrier disruption	Acne severity
Gut	Bacteroidetes *↑/*Firmicutes *↓*	Increased ratio	Metabolic inflammation	Western−diet acne
	Probiotics (*Lactobacillus* spp)	Decreased	Increased systemic inflammation and gut skin microbiota modulation	Acne severity

### 6.1 Skin microbiome-epigenetic interactions

*C. acnes*, long considered the primary pathogen in acne, comprises multiple phylogenetic strains with distinct virulence and immunomodulatory profiles. Multilocus sequence typing studies revealed substantial regional and interpersonal heterogeneity, demonstrating dynamic interactions between *Staphylococcus epidermidis* (*S. epidermidis*) and yeasts such as *Malassezia* species within a complex follicular ecosystem (312–314). Importantly, *C. acnes* is an abundant commensal in both healthy and acne-prone skin, and acne development is linked to strain-level dysbiosis rather than bacterial overgrowth (315).

**Figure 3:**
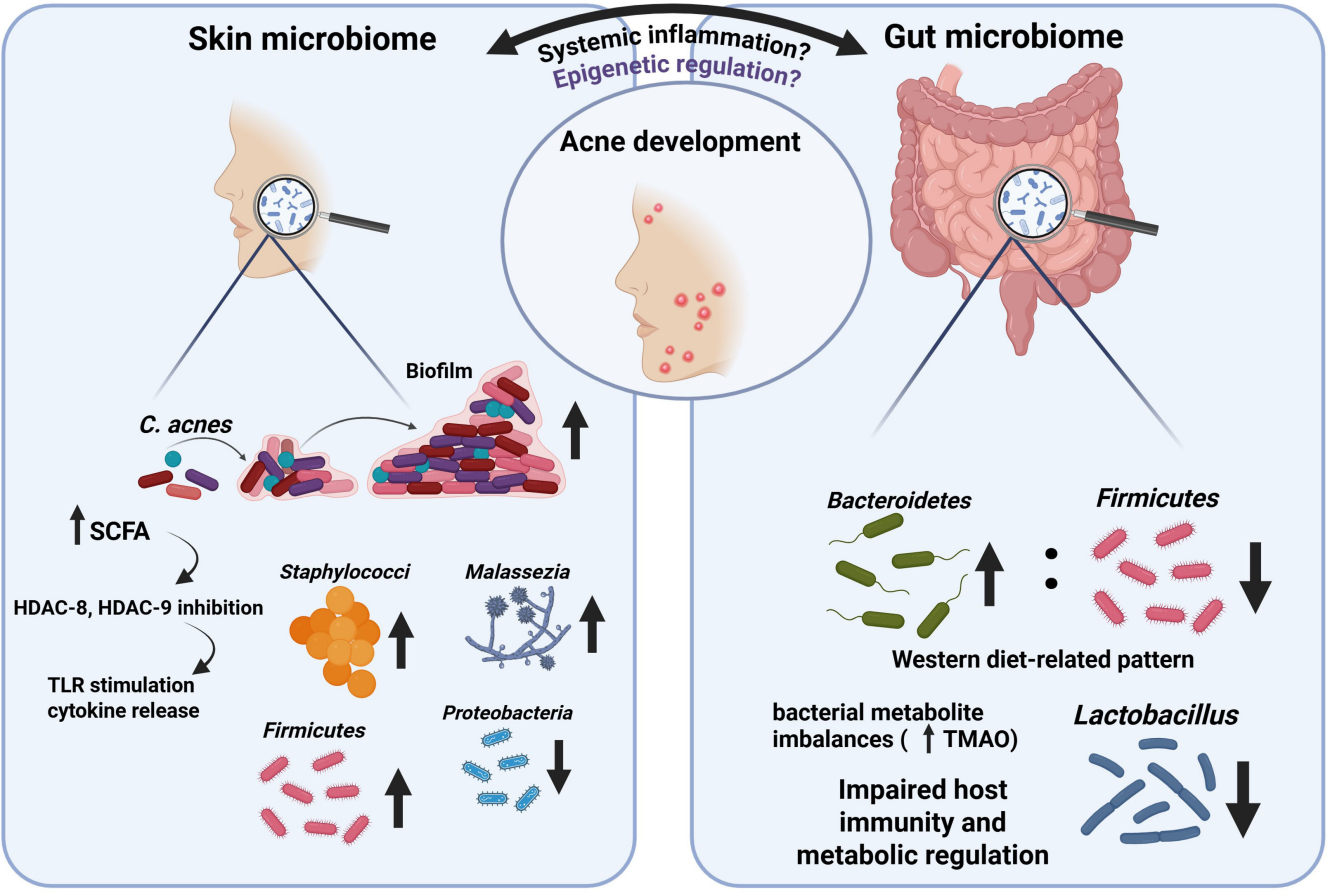
Skin–gut microbiome crosstalk in acne vulgaris. Bidirectional interactions between the skin and gut microbiomes contributing to acne pathogenesis. On the skin, dysbiosis characterized by altered *C. acnes* strain activity, biofilm formation, increased Staphylococci and *Malassezia*, and shifts in major phyla (↑ Firmicutes, ↓ Proteobacteria) promotes innate immune activation. Microbial metabolites, including short-chain fatty acids (SCFAs), modulate host responses through mechanisms such as HDAC-8/HDAC-9 inhibition, TLR stimulation, and downstream cytokine release, amplifying local inflammation. In parallel, gut microbiome alterations associated with Western dietary patterns (↑ Bacteroidetes, ↓ Firmicutes and Lactobacillus) lead to metabolite imbalances (e.g., increased TMAO), impaired metabolic regulation, and systemic immune dysregulation. These gut-derived signals may influence the skin *via* systemic inflammation and epigenetic regulation, ultimately affecting acne development. Arrows indicate relative increases or decreases in microbial taxa or pathways.

Phylotype IA1 is more prevalent in acne lesions, with SLST molecular typing confirming phylotype A1 as significantly more common in acne patients than in controls (316). However, its abundance does not consistently correlate with disease severity, underscoring the importance of microbial functional activity and host epigenetic responsiveness rather than microbial load alone (317, 318). Biofilm formation within pilosebaceous follicles further stabilizes inflammatory niches and contributes to lesion persistence, especially during adolescence (319) (**Figure 3**).

In this context, acne-associated *C. acnes* strains contribute to pathogenesis through the secretion of extracellular vesicles (EVs) which are nanoscale membranous structures that act as potent mediators of host–microbe communication. *C. acnes*–derived EVs induce acne-like phenotypes more rapidly and robustly than whole bacterial extracts (320). EVs activate myeloid cells and their capacity to engage both epithelial and immune compartments. Notably, EV composition varies across *C. acnes* phylotypes, conferring phylotype-specific immunomodulatory potential, with acne-associated strains producing EVs enriched in pro-inflammatory proteins and lipids that induce IL-1β, IL-6, TNF-α, CXCL1/5, and IL-17 (321, 322). These findings reinforce the concept that acne-related dysbiosis reflects qualitative alterations in microbial secretomes, with downstream epigenetic consequences, rather than simple shifts in bacterial abundance.

Microbial metabolites provide a direct mechanistic bridge between the microbiome and the epigenome. Short-chain fatty acids (SCFAs), such as butyrate produced by *C. acnes* under anaerobic conditions, act as endogenous HDAC inhibitors and directly modulate keratinocyte and sebocyte transcriptional programs (323). Experimental inhibition of HDAC8 and HDAC9 enhances Toll-like receptor–mediated cytokine release in keratinocytes and sebocytes supporting the concept that microbial metabolites fine-tune cutaneous immune responses *via* epigenetic mechanisms (324, 325). Disruption of microbiome–epigenome crosstalk predisposes to chronic inflammatory dermatoses, including acne vulgaris (326).

Acne skin shows increased Firmicutes and reduced Proteobacteria relative to healthy controls, with marked genus-level instability. A metagenomic study that identified 7,303 operational taxonomic units (OTUs), 3,833 were unique to acne and 1,011 to healthy controls. Acne-associated taxa include Lachnospiraceae, Clostridiales, Moraxellaceae, *Prevotella*, and *Lactococcus garvieae*, whereas *Achromobacter, Stenotrophomonas, Porphyromonas, Prevotella*, and *Pseudomonas* predominated in controls (327). Longitudinal studies during puberty indicate that these microbial shifts evolves dynamically alongside hormonal and sebaceous changes (328).

Microbial interactions further influence epigenetically regulated inflammation. *S. epidermidis* may exert protective or competitive effects against *C. acnes*, which belongs to phylum Actinobacteria, a dominant component of the healthy skin microbiome, while *Malassezia* species correlate positively with inflammatory severity (329, 330). Clinically, reduced antibiotic efficacy in resistant *C. acnes* strains, indicates that therapeutic benefit derives from microbiome modulation rather than just antimicrobial action (331–333). In a clinical trial of 26 acne patients, both topical erythromycin and a dermocosmetic formulation reduced lesions over 28 days, yet their microbiological effects differed. Erythromycin reduced Actinobacteria, whereas the dermocosmetic decreased both Actinobacteria and Staphylococci, depicting the relevance of microbiome-preserving strategies (334).

### 6.2 Gut microbiome, epigenetic programming, and the gut–skin axis

Systemic microbial dysbiosis further amplifies acne-associated inflammation through the gut–skin axis. Acne patients exhibit higher gut microbial diversity, reduced Actinobacteria, and increased Proteobacteria (327, 335). At the phylum level, decreased Firmicutes and increased Bacteroidetes result in an elevated Bacteroidetes/Firmicutes ratio, a microbial signature linked to Western dietary patterns and low-grade systemic inflammation (336). Within 38 differential taxa, Bacteroidetes, Bacteroidia, and Bacteroidales are enriched in acne. Depletion of beneficial taxa such as Clostridia, Clostridiales, Lachnospiraceae, and Ruminococcaceae, key producers of anti-inflammatory SCFAs, may reduce epigenetic repression of pro-inflammatory pathways in peripheral immune cells (337).

Recent Mendelian randomization studies provide causal evidence linking specific gut microbial taxa to acne susceptibility, supporting a contributory role of gut dysbiosis beyond confounding lifestyle or dietary factors (338, 339). Additionally, altered microbial-derived metabolites further implicate systemic metabolic–epigenetic crosstalk. Elevated circulating trimethylamine N-oxide (TMAO) levels in acne patients suggest enhanced pro-inflammatory signaling and immune priming *via* gut microbiota–host metabolic interactions (340).

Comparative studies also suggest disease-specific gut–skin microbial signatures modulated by demographic and clinical factors. In a study of 8 acne and 19 rosacea patients with matched controls, cutaneous microbial composition was profiled using 16S rRNA gene sequencing of skin samples. The analysis revealed that *Serratia marcescens* and *C. acnes* were more abundant in rosacea lesions compared to acne. Interestingly, *C. acnes* was more prevalent in controls than in patient groups for both conditions, suggesting a potential protective commensal role (341). The *C. acnes* profile in papulopustular rosacea resembles that of acne more closely than non-inflammatory rosacea, indicating overlapping inflammatory microbiome–epigenetic circuits across these dermatoses (19).

Emerging research suggests that gut dysbiosis, or microbial imbalance, may influence acne through systemic inflammation. Probiotics, particularly those containing *Lactobacillus* strains, have shown promise in reducing acne severity by modulating gut and skin microbiota and lowering systemic inflammation (342). Dietary patterns are a key determinant of microbial composition. High-glycemic index and low-fiber Western diets deplete SCFA-producing taxa and enrich pro-inflammatory microbial profiles, promoting systemic inflammation and epigenetic immune reprogramming affecting acne. In this context, probiotic and prebiotic interventions are best considered as nutrition-linked biological modulation that restores the gut-skin axis, rather than as isolated biological therapeutic methods (312, 343).

### 6.3 Microbiome-modulating therapies within the acne exposome

These data position acne as a disease of microbiome-conditioned epigenetic dysregulation, with direct implications for therapy. Probiotic-based interventions in acne vulgaris function as exposure-modifying strategies that modulate host–microbe interactions across dietary, pharmaceutical, and cosmetic domains (344, 345). Oral probiotics, prebiotics, symbiotics, and postbiotics, particularly *Lactobacillus* and *Bifidobacterium* strains, can modestly but significantly reduce acne severity (346–349). They primarily act through the dietary and intrinsic exposome, modulating the gut–skin axis by restoring microbial diversity, attenuating systemic inflammation, improving epithelial barrier function, and influencing metabolic and immune signaling pathways implicated in acne pathophysiology, including IGF-1, mTORC1, and Th17 polarization (221, 346, 350, 351). Randomized controlled trials further support strain-specific efficacy, with *L. plantarum* and *L. paraplantarum* demonstrating anti-inflammatory, antibacterial activity *against C. acnes* and improvements in clinical acne scores (347, 352–357). Additionally, postbiotics and probiotic-derived metabolites as biologically active components exert anti-inflammatory and antibiofilm effects without the risks associated with live organisms (358–360). Novel strategies targeting *C. acnes* virulence, such as antibiofilm compounds and immune-enhancing approaches (e.g., anti-CAMP1 IgG), suggest a shift toward precision microbiome interventions rather than broad-spectrum antimicrobial suppression (361, 362).

In contrast, topical probiotics and postbiotic formulations operate within the cutaneous and cosmetic exposome, directly shaping the local microbial ecosystem, suppressing *C. acnes* while preserving commensal balance (363–365).

Importantly, microbiome-directed strategies may also mitigate iatrogenic exposomal stressors, particularly antibiotic-induced dysbiosis and antimicrobial resistance, which are increasingly recognized as long-term modifiers of acne trajectory (366). Nevertheless, current clinical guidelines acknowledge probiotics primarily as adjunctive therapies, citing heterogeneity in formulations, dosing, and study design as barriers to formal recommendation (5, 96).

## 7 Hormonal and endocrine exposome in acne vulgaris

### 7.1 Cutaneous steroidogenesis as an exposome-responsive system

Human skin is an active site of steroidogenesis, expressing the enzymatic machinery required for the synthesis and metabolism of glucocorticoids, androgens, estrogens, and steroid precursors (367). Sebaceous glands display robust expression of steroidogenic enzymes involved in androgen activation and inactivation, enabling local modulation of the hormonal microenvironment independently of circulating hormone levels (368).

This intracrine steroidogenic activity enables sebocytes to rapidly adapt to internal and external stressors, such as inflammation, metabolic changes, and environmental challenges (369). However, dysregulation of local steroidogenesis may amplify sebaceous lipogenesis and inflammatory signaling, contributing to acne development (367). Environmental stressors and lifestyle-related exposome factors -such as chronic stress, sleep disruption, and metabolic imbalance- may influence cutaneous steroidogenic pathways, indirectly modulating acne severity through endocrine mechanisms (370, 371).

#### 7.1.1 IGF-1 and metabolic hormones linking diet to sebocyte activation

Insulin-like growth factor 1 (IGF-1) represents a key metabolic hormone bridging dietary exposome factors and sebaceous gland activation (372). IGF-1 stimulates sebocyte proliferation, lipogenesis, and inflammation through activation of PI3K/Akt/FoxO1 and JAK2/STAT3 signaling pathways, pathways that are critically implicated in acne pathophysiology (30). Elevated IGF-1 signaling, commonly associated with high-glycemic diets, insulin resistance, and Western dietary patterns, promotes sebaceous gland hyperactivity and creates a pro-inflammatory microenvironment within the pilosebaceous unit (199, 373).

Experimental inhibition of IGF-1–induced signaling attenuates lipid synthesis and inflammation in human sebocytes, supporting diet and metabolism as modifiable exposomal drivers of acne (30).

#### 7.1.2 Retinoid signaling and endocrine modulation of sebocyte function

Retinoids exert their biological effects through retinoic acid receptors (RARs) and retinoid X receptors (RXRs), which function as ligand-activated transcription factors regulating sebocyte differentiation, lipid synthesis, and inflammatory responses (374). Isotretinoin acts indirectly on sebocytes, affecting gene expression, cell differentiation, and metabolic pathways rather than simply inducing sebocyte apoptosis (375). These indirect effects suggest that isotretinoin may modulate, restore dysregulated endocrine and metabolic signaling within the sebaceous gland, counteracting exposome-driven hormonal overstimulation (376, 377).

### 7.2 Acne, obesity, and insulin resistance

Acne vulgaris and metabolic dysregulation seem to be closely associated. In adult males with acne, higher plasma glucose and insulin levels have been observed following oral glucose testing, a correlation not consistently seen in females, suggesting sex-specific metabolic patterns (378, 379). Although body mass index (BMI), waist circumference, and visceral fat area tend to be higher in male acne patients, these differences do not always reach statistical significance (380).

Other investigations on acne patients have documented significantly elevated BMI, body fat percentage, and fat mass among acne patients, with obesity being prevalent. Notably, moderate-to-severe acne is associated with more pronounced metabolic alterations than mild disease, including hyperinsulinemia and metabolic syndrome features (379, 381, 382). Insulin resistance appears more prevalent among acne patients and may represent a predisposing metabolic factor contributing to disease development (383).

In pediatric and adolescent populations, early-onset prepubertal acne (ages 7–12) is significantly associated with higher BMI and increased obesity rates, particularly among females, with prevalence rising with age (384, 385). Hence, metabolic dysregulation and insulin resistance may precede or accompany acne-onset early in life, potentially influencing disease persistence and severity.

### 7.3 Metabolic syndrome and acne severity

The presence of acne scars, often considered a surrogate marker of severe or chronic inflammatory disease, is disproportionately higher among patients with metabolic syndrome. This observation supports a bidirectional relationship between metabolic dysfunction and acne severity, where systemic insulin resistance and oxidative stress may exacerbate inflammatory acne, while chronic acne may reflect underlying metabolic imbalance (386). Furthermore, metabolic assessment and longitudinal follow-up in patients with moderate-to-severe acne may be useful for early detection and management of cardiometabolic comorbidities (380, 387).

Multiple clinical and observational studies demonstrate higher BMI, central obesity, dyslipidemia, and impaired glucose metabolism among acne patients, with metabolic abnormalities being more pronounced in moderate-to-severe disease (387–390). Case series and cross-sectional studies further suggest that acne may represent an early cutaneous marker of systemic metabolic dysfunction, especially in adolescents and young adults (387, 388).

At the molecular level, insulin resistance leads to compensatory hyperinsulinemia and enhanced IGF-1 signaling, resulting in mTORC1 overactivation. This promotes sebocyte proliferation, lipogenesis, and androgen synthesis while suppressing FoxO1-mediated transcriptional control, collectively driving follicular hyperkeratinization and inflammation characteristic of acne (391, 392).

## 8 Social determinants, digital environments, and gender diversity in the acne exposome

The exposome of acne vulgaris extends beyond biological and environmental triggers and includes the social determinants of health (SDOH), the structural conditions shaping exposure, vulnerability, healthcare access, and outcomes across the lifespan. As the “causes of the causes,” SDOH such as socioeconomic position, education, neighborhood environment, healthcare infrastructure, and structural discrimination influence acne onset, severity, treatment trajectories, and psychosocial burden through upstream mechanisms rather than individual behaviors alone (393–395).

Barriers to dermatologic care such as cost, transportation limitations, rural residence, and specialist scarcity, delay diagnosis and treatment escalation, increasing the risk of chronic inflammation, scarring, and psychosocial burden (19, 396, 397). Inequitable access to effective therapies underscores persistent gaps in pharmacoequity, particularly for prescription topical agents, systemic antibiotics, and isotretinoin (398).

Limited access to professional care frequently results in reliance on over the counter (OTC) acne products and informal treatment strategies. Although OTC regimens may provide partial symptom control, prolonged unsupervised use, inappropriate combinations, and variable formulation quality can contribute to irritant dermatitis, suboptimal disease control, and treatment fatigue (106, 399). Socioeconomic and regional disparities strongly shape OTC utilization, particularly in settings where prescription access is delayed or constrained, such as parts of Latin America and Asia (106, 400). Within an exposome framework, self-directed acne care represents a structural consequence of healthcare inequities rather than a purely individual choice.

### 8.1 Digital and social media exposures

Social media platforms represent a pervasive informational and psychosocial layer of the acne exposome. Acne-related content on TikTok and other platforms shows high engagement but inconsistent medical accuracy, frequently promoting anecdotal or potentially harmful practices (400–403). Adolescents and adults with acne commonly report social media as a primary source of treatment information, influencing product choice, expectations, and adherence outside medical supervision (404–407).

Beyond information exposure, social media intensifies appearance-based comparison and stigma, influencing self-presentation, photo-sharing, and dating behaviors, particularly among women and younger individuals (408, 409). Increased exposure to acne-related content is associated with higher appearance anxiety, psychological distress, and social withdrawal, positioning social media as a psychosocial stress amplifier (410–412).

Digital health tools, including teledermatology and online self-management platforms, may improve access and continuity of acne care. However, uneven digital literacy, broadband access, language availability, and device ownership risk reinforcing existing disparities and preferentially benefiting already advantaged populations (413–415). The digital divide thus constitutes a structural exposomal determinant of acne outcomes (416). Equity-oriented deployment is essential for digital acne interventions to avoid widening gaps (417).

### 8.2 Gender diversity, hormonal modulation, and minority stress

Sexual and gender minority populations experience distinct acne-related exposures at the intersection of biological, hormonal, psychosocial, and structural factors. LGBTQ+ individuals face higher rates of healthcare discrimination, delayed care, and mental health comorbidities, potentially exacerbating acne burden and limiting timely treatment (418, 419).

In transgender and gender-diverse individuals, acne commonly emerges or worsens during gender-affirming hormone therapy (GAHT), particularly testosterone-based masculinizing regimens (420–422). A large retrospective matched cohort study demonstrated higher acne incidence and severity in transmasculine individuals initiating testosterone compared with cisgender controls, with the greatest risk during the first year and persistence over time. Increased rates of moderate-to-severe acne requiring systemic therapy were observed. Notably, transfeminine individuals receiving estradiol also showed higher acne incidence than matched cisgender men, indicating risk beyond androgen-dominant regimens (423).

Qualitative and mixed-methods studies highlight acne-related stigma, body image distress, and barriers to affirming dermatologic care in transgender and gender-diverse populations. Acne may interfere with gender expression and carry heterogeneous meanings during GAHT, positioning it as both a biological and psychosocial exposomal outcome within a minority stress framework (424).

Acne in this setting reflects interactions between endocrine modulation, sebaceous gland responsiveness, psychosocial stress, and structural barriers to care. Expert consensus supports anticipatory counseling before GAHT, routine assessment of acne severity and psychosocial burden, and integration of dermatologic care into multidisciplinary gender-affirming pathways (422, 425, 426).

### 8.3 Psychosocial burden as an exposomal outcome

Acne vulgaris is associated with substantial psychosocial morbidity, including anxiety, depression, low self-esteem, body dysmorphic symptoms, and suicidal ideation (427–432). Large-scale and longitudinal studies indicate that acne-related mental health comorbidity is globally prevalent and increasing over time (431). Psychological distress correlates only modestly with objective disease severity; patient-reported outcomes highlight the central role of perceived visibility, stigma, and social evaluation (428, 432).

SDOH critically modifies these relationships. Socioeconomic disadvantage, delayed access to care, and structural marginalization increase the likelihood of prolonged disease duration and cumulative psychosocial harm (430, 431, 433). Racial and ethnic differences further demonstrate socially patterned acne burden, with greater concern regarding post-inflammatory hyperpigmentation and psychosocial impact independent of lesion severity (108).

Acne treatment and mental health interact bidirectionally. Clinical improvement generally enhances psychosocial well-being (432, 434, 435), whereas concerns regarding neuropsychiatric adverse effects, particularly with isotretinoin, need careful patient selection, monitoring, and contextualized risk communication, supported by recent EudraVigilance analyses (436). Resilience, social support, and adaptive coping strategies mitigate adverse outcomes, and adjunctive interventions such as cosmetic camouflage improve emotional well-being without altering disease biology (110, 178, 437).

## 9 Conclusions and future perspectives: towards an equity-oriented acne exposome framework

Acne vulgaris is a chronic, inflammatory disease shaped by cumulative interactions between external exposures, microbial ecosystems, and intrinsic interpatient molecular susceptibility. The exposome framework provides a coherent model to explain disease heterogeneity and variable treatment response, while shifting attention from isolated risk factors to cumulative and context-dependent determinants. Within this framework, the sebaceous gland functions as a central biological sensor of exposomal stress. Recurrent environmental and lifestyle-related exposures induce metabolic, inflammatory, and epigenetic reprogramming that contributes to disease persistence and differential responsiveness to therapy. These mechanisms offer a biological rationale for population-level disparities in acne outcomes (**Figure 1**).

Beyond biological susceptibility, inequities in acne burden are strongly driven by structural barriers to affordable and continuous care. Socioeconomic disadvantage is associated with delayed access to dermatologic services, limited availability of evidence-based treatments due to cost, fragmented follow-up, and greater reliance on non-regulated skincare practices. Health policy analyses consistently demonstrate that such access-related barriers disproportionately affect low-income and underserved populations, reinforcing preventable chronicity and long-term sequelae (438, 439).

Future research and policy efforts should therefore prioritize larger interventional studies and community-level exposomics approaches that integrate environmental exposure data, population health indicators, and healthcare access metrics. The value of scalable, cost-efficient exposome methodologies for identifying high-risk communities and informing targeted interventions without reliance on expensive individual-level profiling is evident (440, 441). Such approaches align exposome science with public health surveillance and equity-oriented policy frameworks (19).

From a health systems perspective, an exposome-informed equity framework supports low-cost, high-impact interventions, including integration of simplified, evidence-based acne management pathways into primary care and community health services, improved access to affordable non-comedogenic skincare, and population-level mitigation of environmental exposures such as air pollution and occupational hazards. At a policy level, incorporating acne management into primary-care clinical guidelines and ensuring reimbursement for essential topical therapies and basic skincare products could represent a pragmatic, equity-oriented strategy to reduce disparities in acne outcomes. Furthermore, promotion of healthy, balanced diets and lifestyle behaviors through educational programs should be incorporated to support overall better wellbeing and long-term disease prevention.

In conclusion, aligning exposome research with healthcare policy and equity principles enables a shift toward more accessible, preventive, and sustainable acne care. Emphasizing affordability, access, and community-level intervention ensures that advances in exposome science translate into meaningful population health gains rather than exacerbating existing inequities in dermatologic care.
